# Personal

**Published:** 1908-12

**Authors:** 


### PERSONAL.

DR. ROSWELL PARK will be tendered a dinner at the Hotel Iroquois, Buffalo, on the evening of December 7, 1908, in commemoration of his twenty-fifth anniversary as a teacher of surgery at the University of Buffalo. A number of distinguished surgeons from other cities have been invited and an unusually interesting occasion is anticipated. Drs. Charles G. Stockton, Charles Cary, Matthew D. Mann, Eugene A. Smith, and H. U. Williams constitute the committee in charge of the event.

DR. GEORGE F. COTT of Buffalo, who, with Mrs. Cott, spent some time abroad during the summer and autumn, has returned and resumed his professional practice at 1248 Main street.

DR. CHARLES A. L. REED of Cincinnati, has received the Order of Chevalier in the Legion of Honor of France. Dr. Reed studied in the hospitals of Paris, is accounted an able student of French, which he speaks fluently, and is president of the Cincinnati Alliance Francaise.

---

DR. CARLOS MACDONALD, of New York, announces his removal to 15 East Forty-Eighth street. Hours: Mondays, Wednesdays and Fridays, 11 to 1. Telephones: 306 38th street, 4254 38th street. Residence and sanitarium, Central Valley, N. Y. Telephone, long distance, No. 6.

---

DR. FREDERIC BRUSH, of Boston, has been appointed Superintendent of the New York Post-Graduate Medical School and Hospital. Before assuming the position he will devote some time to a study of post-graduate instruction and hospital administration in the various American medical centers.

---

DR. and MRS. D. C. EISBEIN returned recently after a four months' tour through Italy and Germany.

